# The Impact of Different Timing Schedules on Prostate HDR-Mono-Brachytherapy. A TCP Modeling Investigation

**DOI:** 10.3390/cancers13194899

**Published:** 2021-09-29

**Authors:** Pavel Stavrev, Nadejda Stavreva, Boriana Genova, Ruggero Ruggieri, Filippo Alongi, Alan E. Nahum, Dobromir Pressyanov

**Affiliations:** 1 Faculty of Physics, “St. Kliment Ohridski” Sofia University, 5 James Bourchier Blvd., 1164 Sofia, Bulgaria; nadejda6557@gmail.com (N.S.); pressyan@phys.uni-sofia.bg (D.P.); 2 Department of Radiotherapy, Specialized Hospital for Active Treatment in Oncology, 1797 Sofia, Bulgaria; boriana.genova@gmail.com; 3 Department of Radiation Oncology, ‘Sacrocuore–Don Calabria’ Hospital, 37024 Negrar, Italy; ruggieri.ruggero@gmail.com (R.R.); filippo.alongi@sacrocuore.it (F.A.); 4 Department of Medical and Surgical Specialties, Radiological Sciences and Public Health, University of Brescia, 25121 Brescia, Italy; 5 Formerly at Physics Department, Clatterbridge Cancer Centre, Bebington CH63 4JY, UK; alan_e_nahum@yahoo.co.uk

**Keywords:** HDR brachytherapy, TCP, hypoxia, resensitization

## Abstract

**Simple Summary:**

Reported clinical data on high dose rate mono brachytherapy of prostate cancer carried out using two different treatment regimens are analyzed in this study. The analysis is based on a mechanistic tumor control probability model, which accounts for a possible increase in the tumor radio-sensitivity during treatment. The aim of the study was to verify a hypothesis that the clinically observed better performance of the longer treatment regimen (28 days vs. 14 days) might be due to a state of initial hypoxia and its ensued overcoming by re-oxygenation and, hence, re-sensitization of the prostate cancer. The performed investigation confirmed the assumption of initially hypoxic stage of the tumor followed by its re-sensitization, thus providing a foundation for the use of prolonged schedules for low- to intermediate-risk prostate cancer treatment.

**Abstract:**

Background: Mechanistic TCP (tumor control probability) models exist that account for possible re-sensitization of an initially hypoxic tumor during treatment. This phenomenon potentially explains the better outcome of a 28-day vs 14-day treatment schedule of HDR (high dose rate) brachytherapy of low- to intermediate-risk prostate cancer as recently reported. Methods: A TCP model accounting for tumor re-sensitization developed earlier is used to analyze the reported clinical data. In order to analyze clinical data using individual TCP model, TCP distributions are constructed assuming inter-individual spread in radio-sensitivity. Results: Population radio-sensitivity parameter values are found that result in TCP population values which are close to the reported ones. Using the estimated population parameters, two hypothetical regimens are investigated that are shorter than the ones used clinically. The impact of the re-sensitization rate on the calculated treatment outcome is also investigated as is the anti-hypothesis that there is no re-sensitization during treatment. Conclusions: The carried out investigation shows that the observed clinical data cannot be described without assuming an initially hypoxic state of the tumor followed by re-oxygenation and, hence, re-sensitization. This phenomenon explains the better outcome of the prolonged treatment schedule compared to shorter regimens based on the fact that prostate cancer is a slowly repopulating tumor.

## 1. Introduction

High dose rate (HDR) brachytherapy and stereotactic body radiotherapy (SBRT) are becoming successful treatment modes for the prostate carcinoma [1]. The probability of local control due to these modes of treatment is high [2,3,4,5,6]. In recent theoretical studies [7,8], the impact on the treatment outcome of prolonged hypo-fractionated schedules applied in SBRT is investigated. The investigations carried out in these works have indicated that it might be beneficial for the treatment outcome in terms of the local control if fractions of irradiations are delivered over a prolonged time interval. These theoretical studies are carried out via tumor control probability (TCP) models accounting for hypoxia and subsequent tumor re-sensitization during the course of the treatment [9,10,11,12]. Hypoxia is one of the reasons associated with poor outcome in prostate cancer treatment [13,14,15,16]. Investigations based on radiobiological modeling of the impact of hypoxia on expected TCP for prostate cancer have been carried out [17,18]. The main ideas about modeling the role of re-oxigenation/resensitization on tumor response to irradiation were advanced by Fisher [19]. In his pioneering work, Fisher [19] outlined several possible mechanisms by which resensitization could be modeled. For instance, in the terminology of Fisher, most of the TCP models which account for tumor resensitization are based on a combination of ‘cell number-dependent’ and ‘non-secular time-dependent’ mechanisms [10,11,12,20,21]. In this work, we use the Zaider-Minerbo TCP model, the derivation of which is based on the approach outlining the dynamics of birth and death processes, while the mechanism of re-oxygenation, which is incorporated in the linear-quadratic (LQ) model of radiation cell kill [7], follows the ideology of ‘secular time-dependent’ mechanism [19].

The development of models accounting for resensitization was induced by the existence of data on animal experiments carried out with different fractionated regimens in which inverse dose behavior was observed [22,23]. The inverse dose behavior means that a better treatment outcome in terms of TCP is achieved with schedules with higher number of fractions delivered in longer treatment time than shorter schedules. It should be stated that this observation is only valid for schedules with a small number of fractions, which is the case of SBRT and HDR brachytherapy. Recent clinical studies emerge which also report a better treatment outcome when a longer treatment schedule is applied. For instance, Alite et al. [24] reported a better outcome of SBRT on non-small cell lung cancer (NSCLC) in five fractions when delivered in 14 days (a Monday–Wednesday schedule) vs five fractions delivered in five consecutive days. Quite recently, a retrospective study on a long-term HDR brachytherapy of prostate cancer treatment outcome was reported by a Swedish single-center study [25], where a better cure rate is observed in the group of patients treated with three fractions in 28 days compared to a group treated with two fractions in 14 days. A similar cure rate (biochemical control) as in the group treated with three fractions is reported in [26] for prostate cancer patients treated with HDR brachytherapy of three fractions in 28 days. In the current study, we analyze the data reported in [25] using one of the TCP models, incorporating a possible increase in tumor radio-sensitivity developed in [7,9].

## 2. Materials and Methods

### 2.1. Data

A retrospective study on a long term treatment outcome is recently reported by a Swedish single-center study [25]. The treatment was a high-dose rate brachytherapy applied as monotherapy for treating low- and intermediate-risk prostate cancer patients. A total of 210 patients were divided into two groups receiving two different treatment regimens. In total, 107 patients were treated with three separate implants (fractions) of 11 Gy each over a four-week period and 103 patients were treated with two implants (fractions) of 14 Gy each over a two-week period. After a median follow-up period of seven years, the following biochemical (prostate specific antigen) failure was found: 5 cases out of 107 patients (4.7%) in the group receiving three fractions and 15 cases out of 103 patients (14.6%) in the group treated with two fractions. The cited failure percentages correspond to a local tumor control of 95.3% in the group receiving three fractions and 85.4% local tumor control in the group receiving two fractions.

Data on the outcome of prostate cancer treatment with HDR brachytherapy applied in three fractions in 28 days (carried out in the Specialized Hospital for Active Treatment in Oncology, Sofia, Bulgaria) with 11 Gy dose per fraction, show a similar cure rate (93.2%). The follow-up time varied from 19 to 81 months, with a median of 45 months. The investigated group of patients consisted of intermediate risk PC patients. Based on it, a range of probable *α*/*β* values was determined in [26], with a most probable value of the *α*/*β* ratio of 4.5 Gy. This value of the *α*/*β* ratio is used in the current study as outlined in Section 2.2.

### 2.2. Data Treatment

It is assumed that HDR brachytherapy is radiobiologically equivalent to external fractionated therapy. Therefore, we apply a mechanistic tumor control probability (TCP) model, accounting for cell repopulation, namely the Zaider-Minerbo model [27], to treat the data. In their work, Zaider and Minerbo solve Kendal’s equation describing the dynamics of birth and death processes [28] and obtain an expression applicable to any temporal protocol of dose delivery. It was previously solved for the case of external fractionated radiotherapy [29] for the general case of different time intervals between the fractions and different doses per fraction:

(1) $$TCP\left(T_{n-1}\right)={\left[1-\frac{S\left(T_{n-1}\right)e^{\lambda T_{n-1}}}{1-S\left(T_{n-1}\right)e^{\lambda T_{n-1}}\sum_{k=1}^{n-1}S^{-1}\left(T_{k-1}\right)\left[e^{\left(-\lambda \right)T_{k}}-e^{\left(-\lambda \right)T_{k-1}}\right]}\right]}^{N_{o}}$$

where *N_o_* is the initial number of tumor cells, *λ* is the cell birth rate, *n* is the number of fractions, *T_n*−1*_* is the total treatment time, *T_k*−1*_* is the time until after the *k*th fraction, and S(*T_k*−1*_*) is the cell survival probability after the *k*th fraction. This TCP model can be used with any model of cell survival probability. In the current study, we use the standard LQ model, disregarding the more complex cell survival models, such as the linear-quadratic-linear (LQL) or the lethal-potentially–lethal (LPL) models, since they have a higher number of parameters. In addition, as pointed out in Ruggieri et al. [30], “the choice of optimal cell-survival model … favors the LQ model”. Thus, the linear-quadratic (LQ) model with complete repair of the sub-lethal cell damage between fractions is assumed, S(*T_k*−1*_*):

(2) $$S\left(T_{k-1}\right)=e^{-\left(\alpha \sum_{i=1}^{k}d_{i}+\beta \sum_{i=1}^{k}d_{i}^{2}\right)}$$

where *d_i_* is the dose of the ith fraction delivered homogeneously and *α* and *β* are the radiosensitivity parameters of the LQ model. We hypothesize that the better treatment outcome of the longer treatment schedule might be due to the initial existence of hypoxic (and hence, radio-resistant) region, which is fully re-sensitized during the longer treatment period. Therefore, we use in this study a modification of the linear-quadratic (LQ) cell kill model, where both radio-sensitivity parameters (*α* and *β*) of the LQ model are assumed to increase in time during treatment, as suggested in [7,9]. We developed in [9] a version of the LQ model where the cell radiosensitivity may increase in time according to the following function:

(3) $$\alpha \left(t\right)=\alpha_{0}e^{-bt^{2}/2}+\alpha_{m}\left(1-e^{-bt^{2}/2}\right)$$

Parameter *b* in Equation (3) determines the rate of re-oxygenation/re-sensitization of the tumor. *α*_0_ is the initial low value of the radiosensitivity of the hypoxic part of the tumor and *α_m_* is the maximum possible value of α reached asymptotically in time. The expression for $\alpha \left(t\right)$ is constructed in such way so that $\alpha \left(t=0\right)=\alpha_{0}\;\mathrm{and}\;\alpha \left(t\to \infty \right)\to \alpha_{m}$. However, it was derived theoretically in [9] based on the assumption of increasing oxygen diffusion from the intracellular medium to the hypoxic regions of the tumor commencing after the start of radiotherapy. The increase is due to the killing and washing way of the oxic more sensitive sub-population of cells. This model was validated through fitting to the experimental dataset of Fisher and Moulder [22].

In a recent work [7], we assumed that *β* is related to *α* through the oxygen enhancement ratio (OER) [20,31] in the following way: $OER=\frac{\alpha_{m}}{\alpha_{0}}=\sqrt{\frac{\beta_{m}}{\beta_{0}}}$, where *β*_0_ is the initially low value of *β* and *β_m_* is its maximum value reached asymptotically in time. We assume that for intermediate times an analogous relation between *α* and *β* should also be valid, i.e., $\frac{\alpha \left(t\right)}{\alpha_{0}}=\sqrt{\frac{\beta \left(t\right)}{\beta_{0}}}$, where:

(4) $$\beta \left(t\right)=\beta_{0}{\left(\frac{\alpha \left(t\right)}{\alpha_{0}}\right)}^{2}$$

Thus, the increase of *β* in time is tied to the increase in α according to Equation (4). Substituting Equations (3) and (4) in (2), in case of equal dose per fraction *d,* one gets:

(5) $$S\left(T_{k-1}\right)=e^{-\left(d\sum_{i=1}^{k}\alpha_{i}+d^{2}\sum_{i=1}^{k}\beta_{i}\right)}$$

where $\alpha_{i}\;\mathrm{and}\;\beta_{i}$ are calculated according to Equations (3) and (4) for the time *T_i_* of the *i*-th fraction.

Therefore, the independent radio-sensitivity LQ model parameters of the modified LQ model are the initially low values of *α* and *β*—$\alpha_{0}\;\mathrm{and}\;\beta_{0}$—and the maximum re-sensitized value of α − *α_m_*. The modified LQ model has an additional parameter, b, determining the sensitization rate.

It should be pointed out that conceptually the approach developed in [7,9] to account for the impact of re-oxygenation on TCP corresponds to a ‘time-dependent’ mechanism of re-sensitization according to the terminology of Fisher [19].

The model is applied for the case of a homogeneous target irradiated homogeneously. A homogeneous target means that the target is homogeneous in cell density (i.e., disease distribution within the prostate is not considered) and in radio-sensitivity. The latter assumption suggests that the whole tumor is initially hypoxic and subsequent synchronous re-sensitization takes place during treatment. Alternatively, if hypoxic regions are identified, we must assume that the tumor is composed of roughly two sub-populations of cells: radio-resistant cells in hypoxic condition and much more radio-sensitive cells in oxic conditions. However, it is demonstrated in [32,33,34] that the radio-sensitive subpopulation has a negligible impact on the treatment outcome so that the outcome is determined primarily by the initially hypoxic component. Therefore, Equations (1)–(5) are applied to describe the behavior of the hypoxic part, which determines the outcome of the whole tumor. The Zaider-Minerbo model used with the modified LQ model is labeled the ZMS (Zaider-Minerbo-Stavreva) model. The sensitization of the tumor in time reflects the possible existence of hypoxia, which is counteracted by re-oxygenation leading to sensitization.

### 2.3. Selecting Values for Some of the Model Parameters

The Zaider-Minerbo model is an individual TCP model. In order to adequately apply it for treating clinical data on a patient cohort comprising different individuals that may differ in their radiobiological parameters, we searched the model parametric space for ranges of parameter values that will satisfy the condition that the average individual TCP of the group treated with two fractions, *TCP_2F_*, is close to the clinically observed value of 85.4% and the average individual TCP of the group treated with three fractions, *TCP_3F_*, is close to 95.3%. We assume that the radio-sensitivity parameters, $\alpha_{0},\;\alpha_{m},\;\beta_{0}$, are normally distributed among the patients, reflecting a possible inter-individual variability of these parameters. We assume in addition that the mean value of $\beta_{0},\;\bar{\beta_{0}}$, is calculated from the mean value of $\alpha_{0},\;\bar{\alpha_{0}}$, as $\bar{\beta_{0}}=\bar{\alpha_{0}}/4.5$, where 4.5 is the most probable value of the *α*/*β* ratio as estimated in [26] for the case of HDR brachytherapy administered in three fractions in 28 days. The range of probable *α*/*β* values was estimated to be 3.5–6 Gy. The search was carried out following the procedure of constructing a distribution of individual TCP values when there is a spread in the individual radio-sensitivity parameters described in [35]. It is demonstrated there that the average individual TCP determined from the individual TCP distribution is also the most probable TCP of the population, estimated as the number of cures to the total number of patients. Therefore, the average individual TCP is also termed the population TCP. In the current study, the initial number of tumor cells was fixed under the assumption of nearly equal size of the treated tumors. The value of the repopulation rate, *λ*, was fixed to 0.02 days^−1^ corresponding to a slowly repopulating tumor such as the prostate. The value of parameter b was also fixed to 0.066 days^−1^ close to an estimate of this parameter obtained in [7,9]. The search was carried out through randomly assigning different values to $\bar{\alpha_{0}},\;\bar{\alpha_{m}},$ their standard deviations $\sigma_{\alpha_{0}},\;\sigma_{\alpha_{m}}$ and the standard deviation of $\beta_{0},\;\sigma_{\beta_{0}}$.

## 3. Results

The following set of parameter values was found to satisfy the imposed conditions, i.e., population $TCP_{2F}≃\sim 85.6\%$ and population $TCP_{3F}≃\sim 95.3\%$:

(6) $$\begin{array}{l} \bar{\alpha_{0}}=0.12;\sigma_{\alpha_{0}}=0.02\;{\mathrm{Gy}}^{-1}\\ \bar{\alpha_{m}}=0.23;\sigma_{\alpha_{m}}=0.02\;{\mathrm{Gy}}^{-1}\\ \bar{\beta_{0}}=\bar{\alpha_{0}}/4.5;\sigma_{\beta_{0}}=0.01\;{\mathrm{Gy}}^{-2} \end{array}$$

for the case of pre-set values of $N_{o},\;\lambda \;\mathrm{and}\;b$, namely:

(7) $$N_{o}=10^{8},\lambda =0.02d^{-1}\;\mathrm{and}\;b=0.066d^{-2}$$

An extra constraint was imposed concerning the *α*/*β* ratio. Since according to the model the *α*/*β* ratio changes in time during treatment, we rejected cases for which the mean in time (*α*/*β*)_i_ ratio was outside the following range: 3.5 < *α*/*β* < 6 Gy. Subscript ‘i’ stands for the *i*-th simulated case.

**Figure 1:**
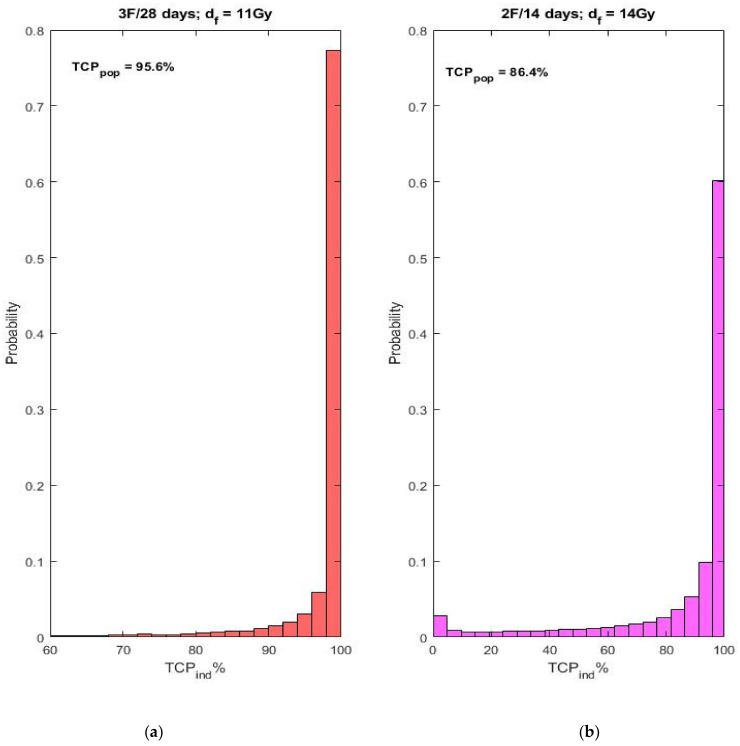
Individual TCP distributions obtained for the parameter values shown in Equations (6) and (7). (**a**)—individual TCP distribution for the three-fraction regimen (5 failures out of 107 patients (4.7%) [25]); (**b**)—individual TCP distribution for the two-fraction regimen (15 failures out of 103 patients (14.6%) as reported in [25]).

The obtained individual TCP distributions for the three fractions in 28 days regimen and for the two fractions in 14 days regimen are shown in Figure 1, subplots a and b, respectively.

**Figure 2:**
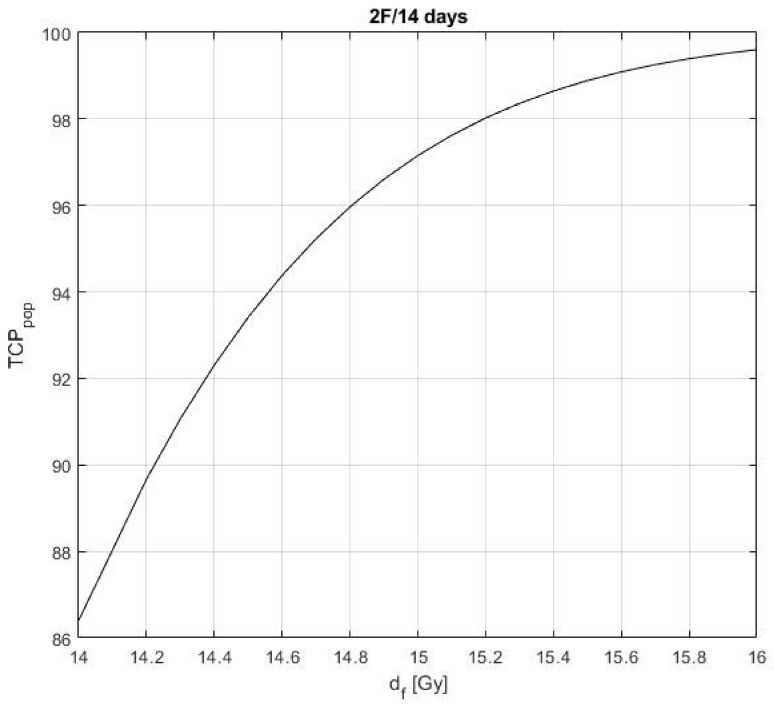
The population TCP in case of the two-fraction regimen, *TCP_2F_*, as a function of the dose per fraction.

After determining the values of $\bar{\alpha_{0}},\;\bar{\alpha_{m}},\;\sigma_{\alpha_{0}},\;\sigma_{\alpha_{m}}\;\mathrm{and}\;\sigma_{\beta_{0}}$ resulting in the clinically observed outcomes, we checked the impact of an increased dose per fraction on the population *TCP_2F_*. The result is shown in Figure 2.

**Figure 3:**
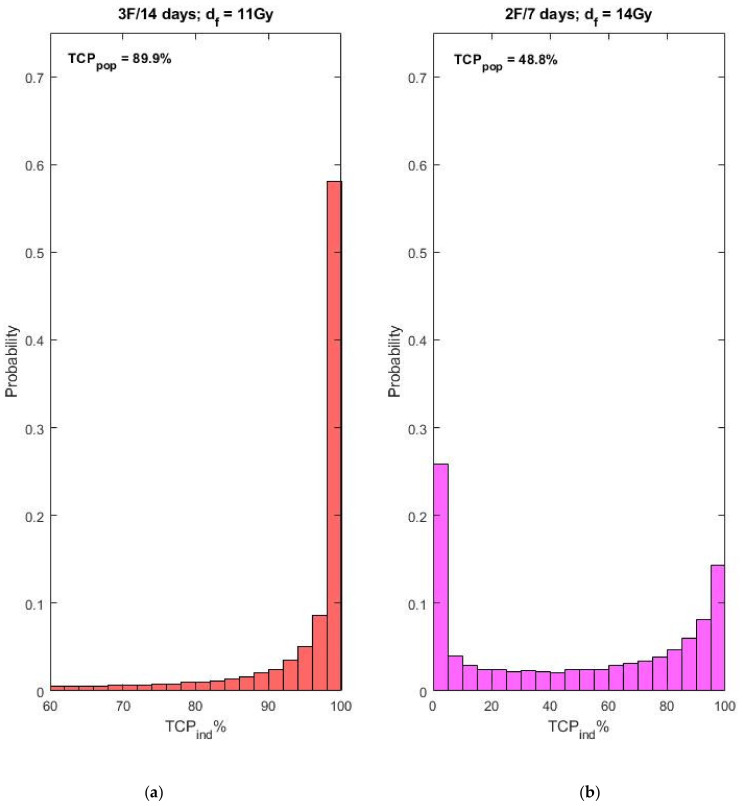
Individual TCP distributions obtained for the parameter values shown in Equations (6) and (7) for shortened schedules. (**a**) Individual TCP distribution for the three fractions in 14 days regimen; (**b**) Individual TCP distribution for the two fractions in 7 days regimen.

We also checked the impact of a shortened treatment time on the treatment outcome for both studied regimens. In the carried out investigation, we kept the number of fractions and the dose per fraction the same as the ones applied in the clinics. However, we assumed a 7-day treatment time in the case of two administered fractions and a 14-day treatment time for the case of three fractions. For both hypothetical regimens, the assumed interval between consecutive fractions was 7 days. The resultant individual TCP distributions are shown in Figure 3. It can be seen that the population *TCP_3F_* now is 89.9% and the population *TCP_2F_* is 48.8%.

The impact of the re-sensitization rate, b, on the treatment outcome is investigated as well using a value of *b* = 1 days^−2^, which determines a much faster process of re-sensitization. (a value of *b* = 1 days^−2^ is obtained as a best-fit value of this parameter by fitting the ZMS TCP model to the animal data of Fowler et al. [23]—this study is in preparation for submission). It turned out that the faster re-sensitization had only a slight impact on the outcome of the clinically applied regimens—the obtained population TCPs are slightly improved ($TCP_{2F}\left|{{}_{b=1d^{-2}}^{14\;days}}_{}\right.=86.7\%$ and $TCP_{3F}\left|{{}_{b=1d^{-2}}^{28\;days}}_{}\right.=95.7\%$). However, this is not a statistically significant difference. For the hypothetical shortened schedules, however, the impact was considerable—the obtained population TCPs are $TCP_{2F}\left|{{}_{b=1d^{-2}}^{7\;days}}_{}\right.=87.9\%$ and $TCP_{3F}\left|{{}_{b=1d^{-2}}^{14\;days}}_{}\right.=96.4\%$, respectively.

In summary, the obtained population TCPs of the prolonged and the shortened schedules for the two assumed values of *b* are:

(8) $$\begin{array}{l} TCP_{2F}\left|{{}_{b=0.066d^{-2}}^{14\;days}}_{}\right.=86.4\%;TCP_{2F}\left|{{}_{b=1d^{-2}}^{14\;days}}_{}\right.=86.7\%;TCP_{2F}\left|{{}_{b=0.066d^{-2}}^{7\;days}}_{}\right.=48.8\%;TCP_{2F}\left|{{}_{b=1d^{-2}}^{7\;days}}_{}\right.=87.9\%\\ TCP_{3F}\left|{{}_{b=0.066d^{-2}}^{28\;days}}_{}\right.=95.6\%;TCP_{3F}\left|{{}_{b=1d^{-2}}^{28\;days}}_{}\right.=95.7\%;TCP_{3F}\left|{{}_{b=0.066d^{-2}}^{14\;days}}_{}\right.=89.9\%;TCP_{3F}\left|{{}_{b=1d^{-2}}^{14\;days}}_{}\right.=96.4\% \end{array}$$

Finally, we investigated the hypothesis that no re-sensitization took place during treatment, i.e., *b* = 0 days^−2^. Two distinct possibilities were examined. First, it was assumed that the initially hypoxic tumor stays hypoxic, i.e., the radio-sensitivities remain equal to their initial values: $\bar{\alpha }=\bar{\alpha_{0}}=0.12;\sigma_{\alpha_{0}}=0.02\;{\mathrm{Gy}}^{-1}\;\mathrm{and}\;\bar{\beta }=\bar{\beta_{0}}=\bar{\alpha_{0}}/4.5;\sigma_{\beta_{0}}=0.01\;{\mathrm{Gy}}^{-2}$. The obtained population TCPs for both clinically used schedules fell to practically zero (1–2%). Second, it was assumed that there was no initial hypoxia and that the tumor was highly sensitive, with radio-sensitivities equal to their maximum values: $\bar{\alpha }=\bar{\alpha_{m}}=0.23;\;\sigma_{\alpha_{m}}=0.02\;{\mathrm{Gy}}^{-1}\;\mathrm{and}\;\bar{\beta }=\bar{\beta_{m}}=\bar{\alpha_{m}}/4.5;\;\sigma_{\beta_{m}}=0.01\;{\mathrm{Gy}}^{-2}$. The obtained population TCP values in this case were around 98% for both clinical schedules.

### Investigating the Impact of Variation in the Parameter Values

**Figure 4:**
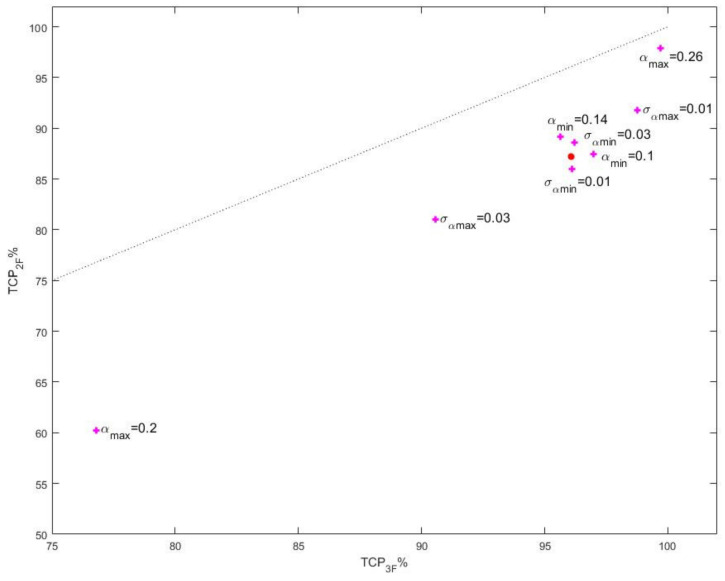
Illustration of the impact of different population radio-sensitivity parameter values on the predicted treatment outcome in terms of population TCP. TCP of the group treated with three fractions—axis X; TCP of the group treated with two fractions—axis Y. The solid red dot corresponds to the population TCPs calculated based on the parameter values from Equations (6) and (7). The varied parameter values are denoted in the figure.

The suggested approach to treating clinical data does not provide a robust procedure for searching and finding values of the population radio-sensitivity parameters that will describe the data. Therefore, we performed an investigation of the sensitivity of the theoretical population TCP values with regard to variation in the population radio-sensitivity parameters. The investigation was carried out via assigning values to the mean and standard deviations of $\alpha_{0}\;\mathrm{and}\;\alpha_{m}$ somewhat differently than the ones given in Equation (6). The results are shown in Figure 4. It can be seen that varying ${\bar{\alpha }}_{0}$ has a smaller impact on the theoretical value of the population TCP than varying ${\bar{\alpha }}_{m}$. Even then, however, the deviation in the theoretical TCP values from the observed ones for both regimens is a few percent, which suggests that the values given in Equation (6) are rather accurately determined.

## 4. Discussion

Based on Figure 2, one can see that with a slight increase in the dose per fraction for the two-fraction regimen from 14 Gy to 14.8 Gy a population *TCP_2F_* of 96% can be achieved. This TCP value is equal to the population *TCP_3F_*. However, side effect estimation, i.e., estimation of complication probabilities of the organs at risk, which may increase due to the higher dose per fraction, is outside the scope of this study.

The results shown in Figure 3 demonstrate that for a slowly repopulating tumor, such as the prostate, shorter regimens than the ones actually applied in the cited clinical study would worsen the outcome in case of low re-sensitization rate. This is especially valid for the two-fraction regimen—an estimated decrease of more than 37% in the population TCP is especially drastic. Only for very high re-sensitization rates, the hypothetical shortened regimens would result in population TCP values comparable to the ones of the clinically applied schedules. The observed independence of the outcome of the prolonged schedules on the value of b can be explained by the assumption that the re-sensitization process will be completed within the treatment time even for low re-sensitization rate. Since, however, the re-sensitization rate is unknown, it can be stated that the prolonged schedules should be regarded as preferable because they are more risk-free resulting in high population cure rates independently from the re-sensitization rate. 

An interesting observation can be made based on Figure 4, further supporting the better performance of the three-fraction schedule, namely that in all examined cases of values of the population radio-sensitivity parameters different than the ones in Equation (6), the calculated TCP values, although different than the clinical ones, are such that always $TCP_{3F}>TCP_{2F}$.

The considerations of the impact of the spread in the radiosensitivity on the individual TCP distribution over the population laid in [35] have indicated that individual TCP distributions could deviate considerably from the normal one and can even exhibit a dichotomous shape. Similar, close to dichotomous distributions are obtained here too—see Figure 1b and Figure 3b, which correspond to the case of two fractions. This means that, although the population (clinically observed) TCP is sufficiently high, there is still a tiny fraction of individuals with close to zero probability of control. It should be noted that dichotomous distribution is not observed in the three-fraction case (see Figure 1a). This theoretical observation may serve as an additional reason for applying a regimen of three fractions in 28 days.

## 5. Conclusions

It is theoretically demonstrated that following the procedure of constructing individual TCP distributions outlined earlier, individual TCP models can be applied to analyze clinical data of prostate HDR-brachytherapy. Moreover, it is possible to extract estimates from these data of the mean values and their standard deviations of the radio-sensitivity parameters of the linear-quadratic model of cell kill (it is also possible to incorporate inter-individual spread in other parameters of the applied TCP model). Consequently, the same procedure can be applied using the estimated model parameters to calculate prospective population TCP values for alternative regimens varying in dose per fraction, time interval between fractions, as well as in number of fractions.

The results of this modeling study show that one possible explanation of the discussed clinically observed treatment outcome is a re-sensitization of an initially hypoxic tumor. Thus, the hypothesis of initial hypoxia in a low- to intermediate-risk prostate cancer and its re-oxygenation during the treatment seems to be validated.

The study also indicates that initial hypoxia and consequent re-oxygenation and, hence, re-sensitization may have positive impact on the outcome of low- to intermediate-risk prostate cancer treatment for very prolonged schedules, utilizing the fact that prostate carcinoma is a slowly repopulating tumor.

## Data Availability

The study did not report any data.
